# Assessing Near-Infrared Spectroscopy (NIRS) for Evaluation of *Aedes aegypti* Population Age Structure

**DOI:** 10.3390/insects13040360

**Published:** 2022-04-07

**Authors:** Teresa Joy, Minhao Chen, Joshua Arnbrister, Daniel Williamson, Shujuan Li, Shakunthala Nair, Maureen Brophy, Valerie Madera Garcia, Kathleen Walker, Kacey Ernst, Dawn H. Gouge, Yves Carrière, Michael A. Riehle

**Affiliations:** 1 Department of Entomology, University of Arizona, Tucson, AZ 85721, USA; tstorch1@email.arizona.edu (T.J.); min2135286@email.arizona.edu (M.C.); arnbrister@email.arizona.edu (J.A.); danielwilliamson@email.arizona.edu (D.W.); lisj@cals.arizona.edu (S.L.); nairs@arizona.edu (S.N.); brophymk@email.arizona.edu (M.B.); krwalker@email.arizona.edu (K.W.); dhgouge@arizona.edu (D.H.G.); ycarrier@ag.arizona.edu (Y.C.); 2 Department of Epidemiology and Biostatistics, University of Arizona, Tucson, AZ 85724, USA; valeriem1@email.arizona.edu (V.M.G.); kernst@arizona.edu (K.E.)

**Keywords:** NIRS, aging, mosquito, parity, SCP1, Sonoran

## Abstract

**Simple Summary:**

Mosquito-borne pathogens require the obligate mosquito vector to shuttle the pathogen between vertebrate hosts. This typically requires the mosquito to acquire the pathogen from an initial bloodmeal, have the pathogen mature and reach the mosquito salivary glands and be transmitted to another vertebrate host in the saliva during subsequent blood feedings. Depending on the pathogen, this incubation period can be up to two weeks. Considering the short lifespan of adult mosquitoes, this means that the oldest mosquitoes are responsible for a disproportionate amount of pathogen transmission. Knowing the age structure of mosquito populations in the field could provide important insights in the likelihood of pathogen transmission occurring. Unfortunately, the current methods of age grading mosquitoes in the field are limited by accuracy, technical challenges and cost. Near-infrared spectroscopy (NIRS) has been shown to be capable of age grading large numbers of mosquitoes cost effectively, although accurate age predictions are still a challenge. In this work, we compared the ability of NIRS to age grade field-collected mosquitoes with two other methods, parity and *SCP1* transcript expression. While we did not find NIRS to be suitable for predicting the precise age of individual field-collected *Aedes aegypti* mosquitoes, we believe that this technique has the potential to monitor changes in the age structure of *Ae. aegypti* populations over time.

**Abstract:**

Given that older *Aedes aegypti* (L.) mosquitoes typically pose the greatest risk of pathogen transmission, the capacity to age grade wild *Ae. aegypti* mosquito populations would be a valuable tool in monitoring the potential risk of arboviral transmission. Here, we compared the effectiveness of near-infrared spectroscopy (NIRS) to age grade field-collected *Ae. aegypti* with two alternative techniques—parity analysis and transcript abundance of the age-associated gene *SCP1*. Using lab-reared mosquitoes of known ages from three distinct populations maintained as adults under laboratory or semi-field conditions, we developed and validated four NIRS models for predicting the age of field-collected *Ae. aegypti*. To assess the accuracy of these models, female *Ae. aegypti* mosquitoes were collected from Maricopa County, AZ, during the 2017 and 2018 monsoon season, and a subset were age graded using the three different age-grading techniques. For both years, each of the four NIRS models consistently graded parous mosquitoes as significantly older than nulliparous mosquitoes. Furthermore, a significant positive linear association occurred between *SCP1* and NIRS age predictions in seven of the eight year/model combinations, although considerable variation in the predicted age of individual mosquitoes was observed. Our results suggest that although the NIRS models were not adequate in determining the age of individual field-collected mosquitoes, they have the potential to quickly and cost effectively track changes in the age structure of *Ae. aegypti* populations across locations and over time.

## 1. Introduction

*Aedes aegypti* (L.) mosquitoes are the principal vectors of several arboviruses of global importance, including dengue, yellow fever and Zika viruses. Efforts to prevent transmission of these viruses typically involve systematic mosquito surveillance focused on either immature or adult life stages. Unfortunately, the associations between the mosquito indices and arbovirus transmission risk are often weak, meaning mosquito density estimates alone may not predict disease outbreaks [1,2]. A mosquito surveillance approach that assessed additional factors related to vectorial capacity besides vector density could be more useful in predicting and preventing arbovirus transmission.

In mosquitoes, the processes of mating, acquiring an infectious blood meal and surviving the extrinsic incubation period account for a large portion of adult lifespan before the pathogen is capable of being transmitted to another vertebrate host. Thus, older mosquitoes are more likely to transmit human pathogens [3,4]. Because of this, the ability to quickly and accurately determine the age structure of wild mosquito populations would be a valuable tool in assessing the efficacy of vector control strategies, understanding the ecology of older mosquitoes and developing models for predicting and responding to mosquito-borne disease outbreaks. While some of this can be accomplished using mark-release-recapture studies, these are limited by logistical and safety concerns and may not be representative of what occurs in wild mosquito populations. In contrast, directly age grading field-collected mosquitoes would eliminate these difficulties and allow researchers to monitor the age structure of mosquito populations over time. Several different techniques have been developed toward this goal, but all face challenges that limit their usefulness.

Historically, parity analysis and the characterization of cuticular hydrocarbons have been used to evaluate the chronological or physiological age of mosquitoes. The Dentinova technique for parity analysis examines tracheal skeins on the surface of the ovary to determine whether a mosquito has successfully completed a reproductive cycle [5]. Intact skeins indicate that the ovaries have not expanded during egg development, and the mosquito has not completed a reproductive cycle, whereas unfurled skeins indicate that a female mosquito has completed at least one gonotrophic cycle. This technique is highly accurate for determining parity, although it does not provide a precise estimate of chronological age because mosquitoes may have completed more than one reproductive cycle or initiated their first reproductive cycle later in life. Polovodova’s method uses the number of dilations in the ovariole follicular tube to estimate the number of reproductive cycles completed [6]. However, this technique is technically challenging and often inaccurate, especially as the number of reproductive cycles increases [7,8]. Recently, a modification to this method using oil injection improved the accuracy of this approach but also increased the processing time and technical difficulty of the technique [9].

The relative abundance of specific cuticular hydrocarbons was shown to be effective at determining the age of individual mosquitoes, first in *Culex quinquefasciatus* Say and later in *Aedes aegypti* [10,11,12]. Subsequently, the ratio of two cuticular hydrocarbons was used successfully to age grade *Anopheles stephensi* females [13]. The drawbacks of cuticular hydrocarbon analyses are that they are time consuming and technically challenging, which limits the number of individual mosquitoes that can be feasibly analyzed.

More recently, considerable work has focused on identifying age-associated genes whose transcript levels change in an age-associated manner. Cook et al. [14] identified ten genes in *Ae. aegypti* whose expression changed in a predictable manner, as the chronological age of a mosquito increased. Using a combination of these genes, they were able to age grade individual mosquitoes with greater accuracy than the cuticular hydrocarbon approach. This work was successfully replicated for age predictions in *Anopheles gambiae*, and a subsequent screen for age-associated genes using microarray analysis identified thousands of transcripts that increased or decreased as a mosquito aged [15,16]. Several of these transcripts changed independent of most physiological events (e.g., nutrition, reproduction). One of the most predictive transcripts for both species was sarcoplasmic calcium-binding protein 1 (*SCP1*), for which the expression decreased in a predictable manner as the mosquitoes aged. We previously utilized this gene to develop an age-grading technique for classifying individual field-collected *Ae. aegypti* mosquitoes into those incapable of transmitting dengue (<5 d), with the potential to transmit dengue (5–14 d) and those at high risk of transmitting dengue (15+ d) [17]. Using this age-grading technique, we were able to determine that *Ae. aegypti* females in a dengue-endemic area of northern Mexico were generally older than females in a nearby region where dengue transmission was rare [18]. Recently, Weeraratne et al. used three age-associated genes, including SCP1, to age grade *Ae. aegypti* and *Ae. albopictus* mosquitoes in Sri Lanka [19]. While this approach is reasonably accurate, its greatest shortcomings include high cost, long processing times and destruction of mosquito samples, all of which limit the ability to screen and validate a large number of mosquitoes.

A different approach for age grading individual mosquitoes, near-infrared spectroscopy (NIRS), uses near-infrared electromagnetic waves to interact with cuticular molecules containing C-H, N-H, S-H or O-H bonds [20,21]. In this technique, the spectra of the head and thorax of adult female mosquitoes are scanned from 350 to 2500 nm and the near-infrared reflectance compared with models developed from the spectra of hundreds of mosquitoes of known age. The advantages of this approach include rapid analysis, minimal cost after the original equipment purchase and non-destructive sampling that allows for validation with other age-grading techniques. Studies in both anophelene and culicine mosquitoes demonstrated that this approach could reliably predict the age of individual mosquitoes from homogeneous populations reared under controlled laboratory conditions [22,23,24,25,26]. However, there are few studies that investigate the efficacy of this approach on field-collected mosquitoes exposed to a variety of environmental and physiological conditions. Studies that address the usefulness of NIRS for individual field-collected mosquitoes have been hampered by a lack of alternative age-grading techniques to validate the approach. For example, a recent study of *Anopheles gambiae* mosquitoes that used parity analysis and the presence of *Plasmodium* sporozoites to identify older mosquitoes could not accurately age grade field-collected mosquitoes using NIRS [27]. Similarly, researchers had limited success using NIRS to age grade *Aedes albopictus* collected as pupae from the wild and reared to adulthood under controlled, semi-field conditions [28]. In this work, we aim to ascertain the usefulness of NIRS for age grading *Ae. aegypti* populations by comparing the NIRS age prediction with results from parity analysis and our previously developed *SCP1* transcript expression analysis [17].

## 2. Materials and Methods

### 2.1. Mosquito Colony Establishment and Maintenance

We used the UGAL strain of *Ae. aegypti* for initial model development. This line was developed from mosquitoes collected at the University of Georgia and has been in continuous culture for nearly 50 years. Mosquitoes were maintained in our ACL2 insectary under a 16 h/8 h day/night cycle at 27 °C and 70% RH. Larvae were reared at a density of 150 individuals per liter of water and fed ground cat chow (Purina Complete, St. Louis, MO, USA). Adults were provided with 10% sucrose ad libitum, and human blood was used for propagating the colony.

We also developed models for a colony of *Ae. aegypti* originally collected in Tucson, AZ, during the summer of 2016 and a second colony established from *Ae. aegypti* collected directly from our experimental sites in Maricopa County, AZ (Phoenix metropolitan area), during 2017. For both colonies, we collected the eggs using simple ovitraps, returned the oviposition substrate (germination paper) to the lab and maintained the eggs under moist conditions for 48 h before allowing them to completely dry. Desiccated eggs were hatched by submerging the egg sheets in deionized water for ~2 h. Hatched larvae were reared to adulthood as described above for UGAL mosquitoes. Tucson mosquitoes were reared in our insectary for four generations and Maricopa mosquitoes for three generations prior to their use in the NIRS model development.

### 2.2. NIRS Model Development and Sample Analysis of Laboratory-Reared Mosquitoes

*Ae. aegypti* mosquitoes used to develop and test the NIRS models were reared under standardized rearing conditions for varying lengths of time following adult eclosion. Non-blood-fed adult females were collected at ages ranging from 1 to 27 days after adult emergence and frozen at −80 °C, mimicking our treatment of field-collected samples. For the UGAL lab model, mosquitoes were reared under controlled conditions (27 °C; 70% RH) in the insectary until the appropriate age. For the UGAL semi-field model, lab-reared UGAL adult females were collected within one day of adult emergence and subsequently maintained outdoors in 2.5-L cages (~100/cage) during May in Tucson, AZ, under shaded conditions to represent a typical exposure in southern Arizona. Finally, our Tucson and Maricopa NIRS models were based on females from our recently established colonies (F4 for Tucson colony and F3 for Maricopa colony) reared in our insectary to known ages and frozen at −80 °C upon collection.

**Figure 1:**
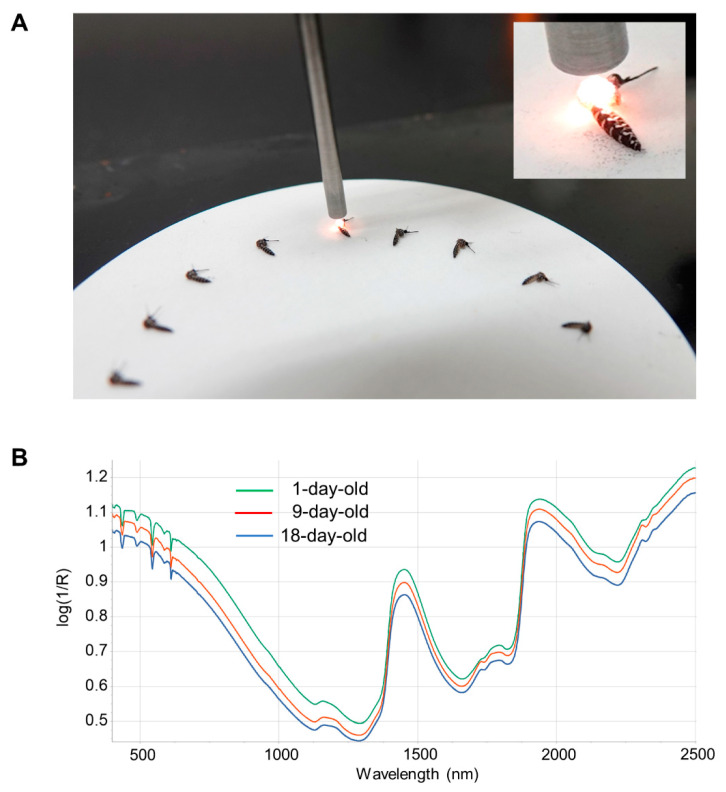
NIRS scanning and typical spectra. (**A**). Examples of mosquito positioning under the NIRS probe. The NIRS probe was positioned 3 mm above the Spectralon plate and ~1.5 mm above the mosquito thorax. (**B**). Average log(1/R) spectra from 1- (green line; *n* = 53), 9- (red line; *n* = 53) and 18-day-old (blue line; *n* = 60) mosquitoes. Wavelength across the visible and near-infrared spectrum is indicated on the X-axis.

Before NIRS scanning, mosquitoes had their legs and wings removed to standardize mosquito positioning and improve the consistency of the NIRS scan (Figure 1). Frozen mosquitoes were thawed at room temperature prior to scanning. Mosquitoes were placed on a 10 cm Spectralon plate and scanned with an ASD LabSpec 4 standard resolution spectrometer (Malvern Panalytical, UK) using a bifurcated fiberoptic probe with six illumination fibers and a single collection fiber. The probe was consistently positioned 3 mm above the Spectralon plate and approximately 1.5 mm above the mosquito (Figure 1). Spectral curves from 350 to 2500 nm were collected 50 times and averaged for each mosquito. After the spectra were acquired and averaged, the ovaries were isolated for parity analysis and the head/thorax used for total RNA isolation (see below).

The acquired spectra were analyzed using the Grams IQ™ software package (Thermo Fisher Scientific, Waltham, MA, USA). Models were developed for the following four treatments: UGAL lab, UGAL semi-field, Tucson and Maricopa. Models were generated using a partial least-squares regression (PLS) in the GRAMS IQ™ spectroscopy software package (Thermo-Scientific, Waltham, MA, USA). A training set was assembled using 80% of the samples arbitrarily selected from the total sample set, with the remaining 20% used to validate the model. Multiplicative scatter correction (MSC) was performed on all models to account for the variation in spectral pathlengths and Savitzky–Golay derivative to linearize the spectra. As reported in previous studies, the visible spectrum (350–700 nm) had considerable noise and did not contribute to age identification, thus it was partially (UGAL lab model) or completely (UGAL semi-field, Tucson and Maricopa models) excluded from all models. Additionally, the UGAL lab and semi-field models were improved by excluding a central portion of the spectrum (Table 1). The final models used 10 to 14 factors and had R^2^ values between 0.66 and 0.79. Details of the final model parameters can be found in Table 1.

### 2.3. Field Collection of Aedes Aegypti Mosquitoes

**Figure 2:**
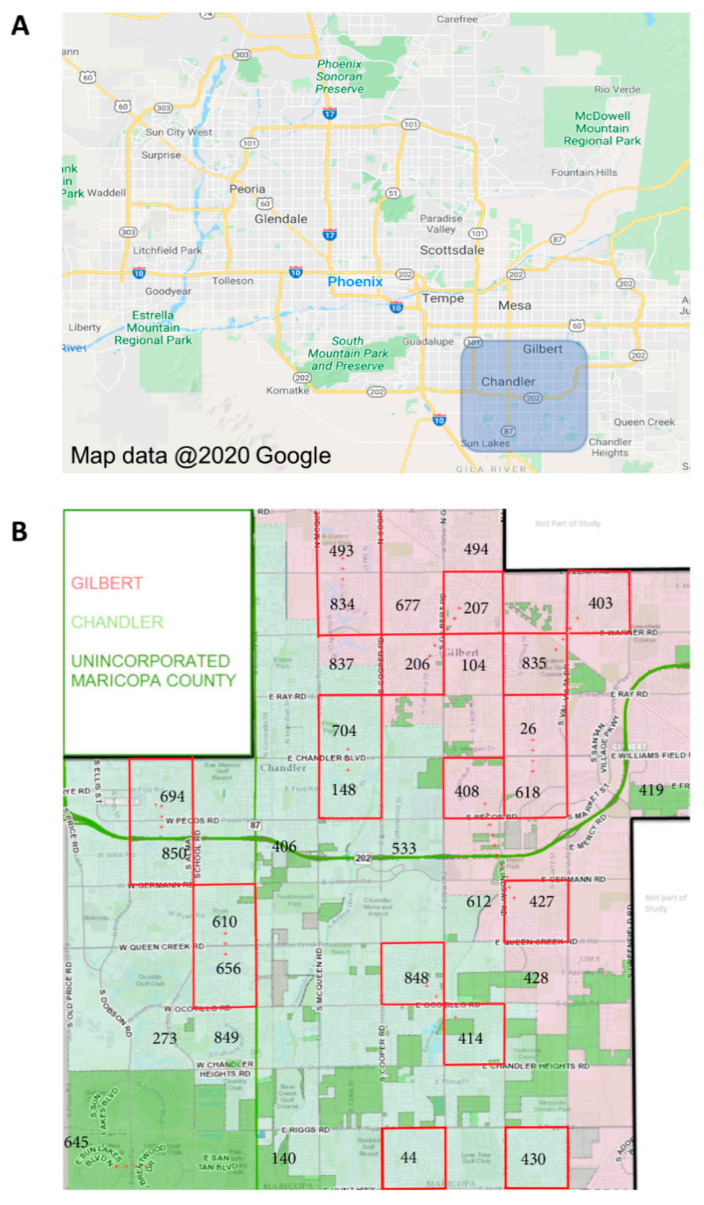
Location of field collections of *Ae. aegypti* mosquitoes in Maricopa County, Arizona. *Aedes aegypti* mosquitoes were collected during the monsoon seasons (approximately July through October) in southeastern Maricopa County and included the cities of Chandler, AZ, and Gilbert, AZ (**A**). Mosquitoes were collected from 20 paired collection sites (**B**) using BG sentinel traps (3 traps distributed per site) for one night each week throughout the collection period. A total of 201 female *Ae. aegypti* in 2017 and 172 in 2018, representing ~15% of the total number of females collected, were used for parity analysis and *SCP1*/NIRS age predictions.

*Ae. aegypti* mosquitoes were collected from Gilbert, AZ, and Chandler, AZ (suburbs of Phoenix, AZ, in southern Maricopa County), during the monsoon seasons (approximately July through October) of 2017 and 2018 (Figure 2A). These mosquitoes are hereafter referred to as “field-collected mosquitoes”. A total of twenty study blocks, each one square mile, were established. Mosquito trapping sites were established at three residences in each study block, each separated by 0.5km (Figure 2B). Adult *Ae. aegypti* mosquitoes were collected using BG sentinel traps (3 per study block; 60 traps total) that were set out overnight (average of 18 h) once per week from July to October of each year. Live mosquitoes were sorted to species, and female *Ae. aegypti* mosquitoes were immediately frozen at −80 °C to inhibit RNA degradation. Frozen samples were transported on dry ice to the University of Arizona, where they were maintained at −80 °C until age-grading analysis.

### 2.4. Parity Analysis of Field-Collected Mosquitoes

The parity status of a subset of the field-collected female *Ae. aegypti* was determined by examining the ovaries for the presence or absence of tracheal skeins per Dentinova [5]. Mosquitoes were arbitrarily selected for parity analysis from all trap collections based on total trap count with a maximum of 10 individuals from any single trap. Following NIRS scanning, the abdomens of field-collected mosquitoes were dissected in saline buffer (128 mM NaCl, 4.7 mM KCl and 1.9 mM CaCl2). The ovaries were gently lifted from the exposed abdominal cavity to avoid stretching of the tracheal skeins and placed onto a clean glass slide. The ovaries were allowed to air dry until they adhered to the slide and then were scored at 400× magnification with a Nikon compound microscope. The ovaries with intact tracheal skeins were considered nulliparous, and those with stretched and tangled ovarian trachea were scored as parous. Mosquitoes with residual blood in the midgut or with vitellogenin deposits in developing ovarioles were considered parous.

### 2.5. SCP1 Transcript Analysis of Field-Collected Mosquitoes

To validate the predicted age of NIRS scanned mosquitoes, we examined the transcript expression of a key age-associated gene, *SCP1*. Previously, we demonstrated that *SCP1* expression decreases as *Ae. aegypti* mosquitoes age in a predictable manner, allowing us to classify individual, field-collected mosquitoes as non-vectors (0–5 days old), unlikely vectors (6–14 days old) and potential vectors (15+ days old) [17]. More recently, we developed a regression model for *SCP1* expression to age grade these mosquitoes more accurately. We used an ordinary least-squares (OLS) regression model to relate *SCP1* expression levels to log-transformed ages (in days) (R package rms; Harrell, 2016) using adult *Ae. aegypti* with known ages (laboratory and semi-field) [17]. A three-knot restricted cubic spline was used to model the non-linear relationship between *SCP1* expression and age. This model enabled the prediction of mosquito age using transcription data in their continuous form. Following parity analysis, the heads and thorax of individual parous mosquitoes were homogenized in RLT buffer and total RNA isolated using the RNeasy Total RNA kit (Qiagen, Valencia, CA, USA). Nulliparous mosquitoes were not assayed, since our previous study demonstrated that they all scored less than 5 days old [17]. Total RNA was converted into cDNA using the High-Capacity cDNA Reverse Transcription Kit (Thermo Fisher Scientific, Waltham, MA, USA). *SCP1* and *RPS17* (controls) transcript titers were determined using qPCR as previously described [17]. The resulting Ct values relative to *RPS17* controls were used in the *SCP1* linear regression model to calculate the age of individual mosquitoes.

### 2.6. Statistical Analysis

For mosquitoes collected in Maricopa in 2017 and 2018, a Kruskal–Wallis ANOVA followed by a Dunn’s post hoc test were used to compare NIRS age predictions from each of the four NIRS models (i.e., UGAL lab, UGAL semi-field, Tucson and Maricopa) between the parous and nulliparous mosquitoes. To evaluate the association between the age predicted by *SCP1* analyses and each of the four NIRS models, we first used simple linear regression. After removing observations with Studentized residuals > |2| corresponding to unexpectedly low or high NIRS values, we used simple linear regression again to estimate the slope, intercept and their associated 95% confidence interval for analyses involving each NIRS model. Although the accuracy of *SCP1* analyses remains unclear, a comparison of these slopes and intercepts provides relevant information on the relative accuracy of the NIRS models. Specifically, NIRS models with slopes close to 1 would have age predictions similar to *SCP1*, whereas NIRS models with lower slopes would tend to underestimate age relative to *SCP1*. Furthermore, NIRS models with intercepts not significantly different from 0 would have similar age predictions as *SCP1* for young mosquitoes (and older ones if the slope does not differ from 1), whereas NIRS models with intercepts greater than 0 would tend to overestimate the age of young mosquitoes relative to *SCP1* (and of older ones if the slope is not different from 1). For each year, we used the overlap of the 95% confidence intervals with 1 and 0 for the slope and intercept, respectively, to compare age predictions between the NIRS models and *SCP1*. We also used the overlap of the 95% confidence intervals associated with the slopes to compare the relative accuracy of the NIRS models.

## 3. Results

### 3.1. Generation of the NIRS Age-Grading Models

**Figure 3:**
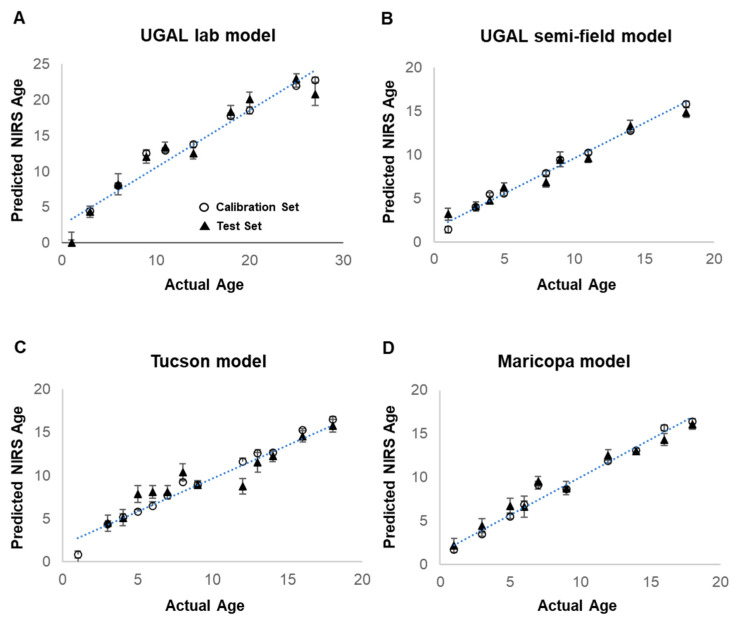
Development of four models for NIRS age grading of *Ae. aegypti* mosquitoes. Predictive NIRS models for the age of female *Ae. aegypti* were generated for four different treatment groups of laboratory-reared mosquitoes. The UGAL lab model (**A**) was generated from 770 laboratory-reared UGAL female *Ae. aegypti* maintained at 27 °C and 70% RH. The UGAL semi-field model (**B**) also utilized the UGAL line but maintained 748 adult mosquitoes in shaded field cages in Tucson, AZ. The Tucson model (**C**) was generated from 540 female *Ae. aegypti* from an F4 line established from eggs collected in Tucson, AZ, and maintained under laboratory conditions. Finally, the Maricopa model (**D**) was generated from 475 female *Ae. aegypti* using an F3 line established from *Ae. aegypti* eggs collected at our actual collection site in southeastern Maricopa County and maintained under laboratory conditions. For all models, open circles represent the calibration set and show mean predicted age at each timepoint of all mosquito spectra used in the model calibration. Solid triangles represent the test set and show the mean age prediction from mosquito spectra withheld from the model calibration and used to validate the model. The number of mosquitoes utilized for the prediction and test set, the spectral range and bend regions selected from the model, the number of factors and other parameters describing each model are shown in Table 1.

Female *Ae. aegypti* from laboratory-reared colonies (UGAJ, UGAL lab and UGAL semi-field models; Tucson lab colony; or Maricopa lab colony) were collected at known ages ranging from 1 to 18 days post-adult eclosion for each model (1 to 27 days for the UGAL lab model). Total sample sizes for each model ranged from 475 to 770 (Table 1; breakdown of daily n in Supplemental Table S1). All four NIRS models predict the age of laboratory-reared mosquitoes reared under controlled laboratory conditions with a reasonable degree of precision, as determined by R^2^ values ranging from 0.66 to 0.79 (Figure 3; Table 1). The Maricopa model has a slope and intercept that are not different from 1 and 0, respectively (based on the 95% confidence intervals), showing that it is an accurate model for estimating the age of laboratory-reared *Ae. aegypti*. The UGAL lab, UGAL semi-field and Tucson models have slopes significantly lower than 1, indicating that they underestimate the age of mosquitoes. Furthermore, their intercepts are significantly greater than 0, showing that they overestimate the age of newly hatched mosquitoes by about one day (Table 1).

The mean age predictions of the test set (solid triangle), consisting of lab-reared mosquitoes withheld from model development, are close to the calibration set used to generate the model. However, considerable variation in the predicted age of individual mosquitoes selected for validation occurs across known ages for all models (Supplemental Figure S1). The final models have ratio of performance deviation (RPD) values of 1.70 to 2.18, with the Maricopa model having the most successful calibration. RPD values are the ratio of the standard deviation of the test set and the standard error of prediction (SEP) [29], with RPD values above 1.5 being suitable for high/low classification and above 2.0 for course quantitative prediction [30].

### 3.2. Parity Analysis of Field-Collected Aedes Aegypti Mosquitoes and a Comparison to NIRS Models

**Figure 4:**
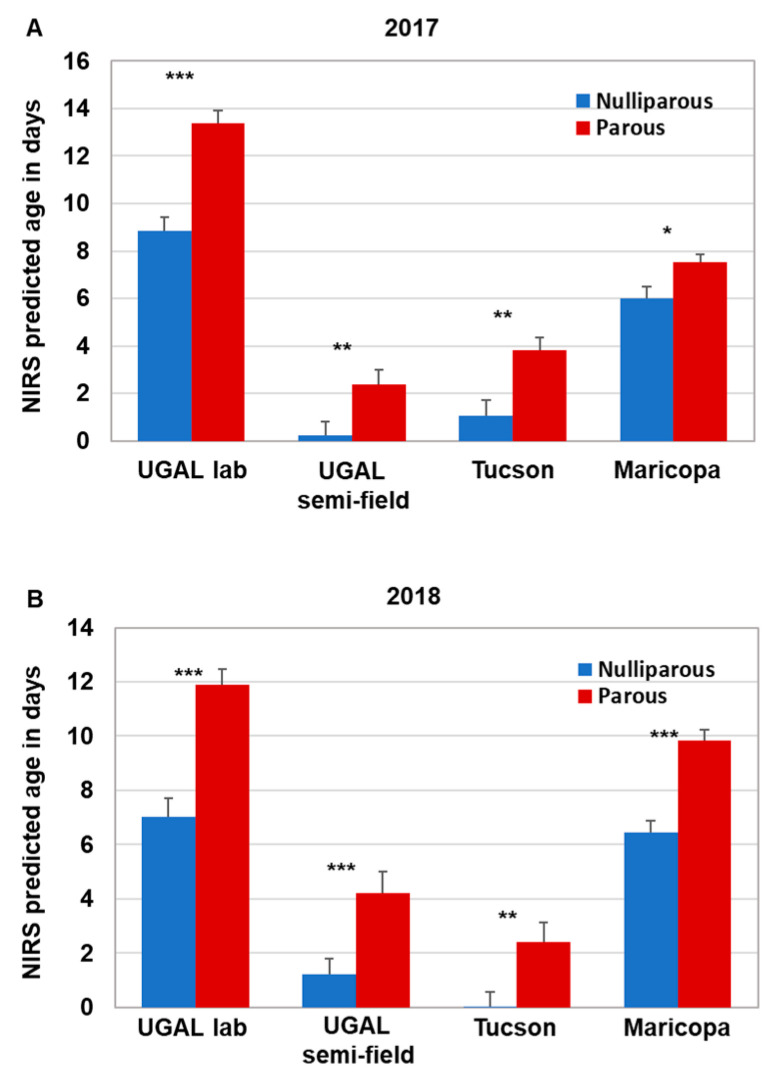
Parity status of field-collected mosquitoes relative to NIRS age predictions. Female *Ae. aegypti* from our field-collected samples in 2017 (**A**) and 2018 (**B**) were separated based on parity status. NIRS spectra for field-collected nulliparous and parous mosquitoes were acquired, and NIRS age predictions for the four models were determined. As expected, field-collected parous mosquitoes were scored significantly older than nulliparous mosquitoes by each of the NIRS models. Significance for each pairing was determined using the Kruskal–Wallis ANOVA followed by a Dunn’s post hoc test (significance is only shown between parous and nulliparous comparisons * = *p* < 0.05; ** = *p* < 0.01, *** *p* < 0.0001).

Parity analysis was conducted on a subset of the field-collected mosquitoes collected monthly during the 2017 (*n* = 201) and 2018 (*n* = 172) monsoon season in Gilbert and Chandler, AZ. This subset represents approximately 15% of the total number of female *Ae. aegypti* collected. Overall parity levels ranged from 49 to 82% during the monsoon seasons of 2017 and 2018. Our previous work using the SCP1 transcript to age grade field-collected *Ae. aegypti* suggested that the majority of nulliparous mosquitoes were less than 5 days old, although the age of parous mosquitoes varied widely [17]. Thus, we compared the parity status of individual field-collected mosquitoes with the NIRS age prediction across the four models to determine whether the NIRS age predictions conformed to parity status (i.e., nulliparous mosquitoes are young, and parous mosquitoes are older; Supplemental Data S1). Indeed, the average age of field-collected mosquitoes scored as nulliparous was significantly less than parous mosquitoes in both 2017 and 2018 across all four models (Figure 4). The actual predicted ages differed significantly between most models, with the exception of the 2017 UGAL semi-field/Tucson and 2018 UGAL lab/Maricopa models, due to variation in model construction. However, the significantly lower age of nulliparous mosquitoes across all models supports the idea that each model could be useful to assess relative changes in the population age structure over time.

### 3.3. SCP1 Analysis of Field-Collected Aedes Aegypti Mosquitoes and Their Comparison to NIRS Models

**Figure 5:**
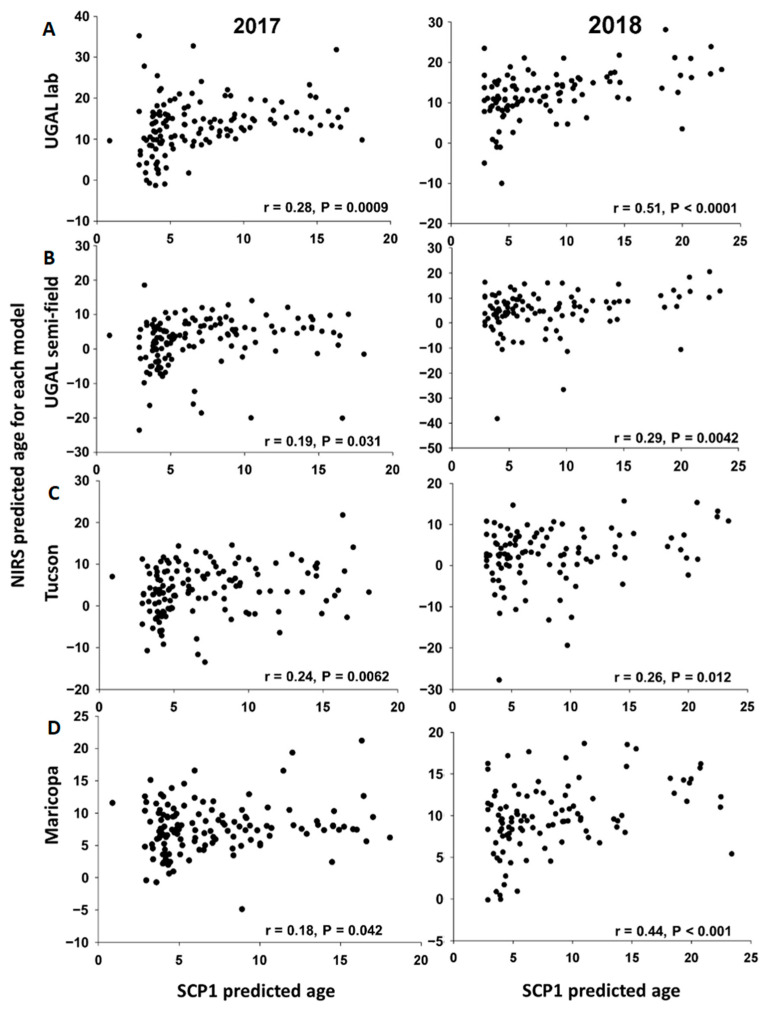
Scatterplots comparing the age of individual field-collected female *Ae. aegypti* predicted with SCP1 or the four NIRS models. For 2017 and 2018, the scatterplots show the relationship of individual age predictions based on SCP1 (X-axis) and each of the NIRS models (Y-axis). (**A**). UGAL lab, (**B**). UGAL semi-field, (**C**). Tucson and (**D**). Maricopa models.

SCP1 expression levels were determined for all parous mosquitoes from the subset used for parity analysis (*n* = 236) and used to estimate the age of individual field-collected mosquitoes. The scatterplots for the associations between age predicted by SCP1 analyses and the four NIRS models are shown in Figure 5. For each model and year, some observations have unexpectedly low or high NIRS values, as revealed by Studentized residuals > |2|. After removing these extreme values, we estimated the slope and intercept of the association between age predicted by SCP1 and each of the four NIRS models (Table 2). With the exception of the Maricopa model in 2017, all slopes are significantly greater than 0, indicating a positive linear association between ages estimated with SCP1 analyses and the NIRS models. With the exception of the Maricopa model in 2017, the slopes do not differ significantly from each other, indicating that the NIRS models provide comparable estimates of mosquito age relative to SCP1. However, for all NIRS models, the slope is significantly lower than 1, indicating that the NIRS models tend to underestimate the age of older mosquitoes relative to the age estimated with SCP1 analyses (Table 2). Furthermore, the intercept is significantly greater than 0 for some models (notably the UGAL lab and Maricopa models), indicating that such models overestimate the age of young mosquitoes relative to results from SCP1 analyses (Table 2).

## 4. Discussion

NIRS has considerable promise for age grading large numbers of mosquitoes quickly, cost effectively and non-destructively [22]. While this approach has proven effective for a number of different mosquito species under controlled laboratory conditions, it has provided mixed results for field-collected mosquitoes [27,28]. In this study, we used results from two well-established age-grading techniques, parity analysis and *SCP1* transcript expression, to assess how four different NIRS models performed in evaluating the age of field-collected *Ae. aegypti* female mosquitoes. The four models generated from independent mosquito colonies reared under specific adult conditions were relatively precise (i.e., high R^2^ values) in predicting the average age of groups of mosquitoes reared under the same conditions, although three of the four models slightly overestimated the age of young mosquitoes (i.e., intercept > 0) and tended to underestimate the age of older mosquitoes (i.e., slope < 1) (Table 1). The effectiveness of these models is not surprising, as the mosquitoes used to train and validate the models were reared under identical conditions. Yet, even under these homogeneous rearing conditions, considerable variation in the predicted age of individual mosquitoes was observed (Supplemental Figure S1). We did find that NIRS was generally suitable for age grading field-collected mosquitoes, as each of the four models consistently graded parous mosquitoes as significantly older than nulliparous mosquitoes, and a significant positive association occurred between *SCP1* age predictions and NIRS age predictions for seven of the eight analyses performed over two years. Nevertheless, the accuracy of the models differed significantly, as shown by the variation among models in the predicted age of nulliparous and parous mosquitoes. Furthermore, relative to age determined from *SCP1* analyses, all NIRS models had a tendency to underestimate the age of older mosquitoes, and some models significantly overestimated the age of young mosquitoes (Table 2). This is likely due to the fact that field-collected mosquitoes developed under different environmental conditions or potentially expressed genetically based differences in their cuticular structure relative to the mosquitoes used to train the models. It is important to note that this limitation is inherent in any age-grading technique, including *SCP1* transcript analysis, which is based on laboratory-reared mosquitoes for their baseline data.

While NIRS may not be ideal in predicting the age of individual field-collected mosquitoes, we suggest that it is valuable for monitoring the overall age structure of an *Ae. aegypti* population as it changes over time. One of the greatest strengths of NIRS is the ability to rapidly screen large numbers of mosquitoes cost effectively, after the initial expense of the NIRS equipment [22]. Parity and transcript analysis are technically challenging, time consuming and expensive, meaning that only a subset of collected mosquitoes can be screened. In contrast, it is entirely feasible for all collected *Ae. aegypti* to be screened with NIRS, since the technical requirements and time required to collect the spectra are minimal. Based on our comparisons between NIRS age predictions and parity status, the difference in age was remarkably robust throughout the study. Regardless of the model or year, nulliparous mosquitoes were consistently aged 3–4 days younger than parous mosquitoes. However, the estimated age of nulliparous and parous mosquitoes varied across NIRS models, with the UGAL lab and Maricopa models generally indicating older ages than the UGAL semi-field and Tucson models. Based on previous results from *SCP1* analyses indicating that most field-collected nulliparous mosquitoes were less than 5 day old [15], it appears that the UGAL lab model overestimated the age of mosquitoes. This suggests that NIRS could be used to assess relative changes, including how the population age structure fluctuates throughout a typical transmission season or how effectively control measures impact the local mosquito age structure following treatment. Compared with other age-grading techniques, the variability of these data would be mitigated by the larger sample size, assuming that sufficient numbers of mosquitoes can be collected. Equally important, the non-destructive nature of NIRS allows for validation with any current or future age-grading techniques.

Of the variations found in field-collected mosquitoes discussed above, perhaps the easiest to account for is genetic diversity. Of our four models, two of them, UGAL lab and UGAL semi-field, were generated using long-established lab colonies of *Ae. aegypti*. The other two used recently colonized mosquitoes originally collected directly from the collection site (Maricopa) or from a population in a similar environment approximately 100 miles away (Tucson). Our assumption was that the models generated from recently collected mosquitoes directly from the study site would more accurately represent the genetic diversity of the local mosquito population. When comparing NIRS to parity, we were surprised to find a high level of consistency in the precision of the models as described above, although considerable bias was observed, particularly in the UGAL semi-field and Tucson models, which predicted both nulliparous and parous mosquitoes as likely too young for their physiological status (i.e., <1 d for nulliparous and <4 d for parous). Furthermore, when we compared NIRS and *SCP1* age predictions of individual mosquitoes, we again did not discern noticeable differences in bias (i.e., difference among slopes) between the Maricopa model and others, and two models (UGAL lab and Maricopa) notably overestimated the age of young mosquitoes (i.e., had intercepts > 0). The fact that the Maricopa and Tucson models did not perform better than the UGAL lab and UGAL semi-field models does not support the hypothesis that genetic similarity could improve the accuracy of the NIRS models. Other variables, such as nutrient availability and environmental variation, are more difficult to control for, since models cannot be developed to account for all of these potential variations during mosquito development, but again, the NIRS approach has the advantage of potentially large sample sizes to mitigate these variations and their impact on the accuracy of predictions. Furthermore, advances in machine learning have the potential to improve these models over time. For example, Milali et al. have utilized artificial neural networks to improve the accuracy of age grading mosquito populations, and we are exploring these approaches for future studies [31,32].

Of the two additional methods we used to estimate the age of field-collected mosquitoes compared to the NIRS predictions, parity is the most accurate. A person skilled in ovary dissections can correctly assess parity nearly 90% of the time, although care must be taken not to stretch the ovaries and unravel some or all the skeins on the ovary [8]. In contrast, both the *SCP1* and NIRS age-grading techniques are known to have varying degrees of bias and random error. Previously, we demonstrated that *SCP1* age grading could correctly categorize mosquitos into one of three groups (<5 days, 6–14 days and >15 days) ~90% of the time [17]. However, in our current study, a number of parous mosquitoes were scored as less than 5 days old (Figure 5) using the *SCP1* technique, which, although possible, is unlikely taking into account the time needed to find a mate, acquire a bloodmeal and complete a reproductive cycle. For NIRS, our work and others’ have consistently found that the oldest mosquitoes are typically scored younger than their actual age [33]. Thus, a variation in the accuracy of both the *SCP1* and NIRS age predictions likely accounts for the discordance observed when comparing these two approaches. In all cases, however, the *SCP1* age predictions are significantly positively associated with the NIRS age predictions, again suggesting that NIRS can be useful to characterize changes in age structure at the population levels if sufficient samples are available.

In summary, we developed a variety of NIRS models to attempt to age grade *Ae. aegypti* mosquitoes collected from the field. NIRS predicted the age of parous mosquitoes as significantly older than nulliparous mosquitoes, as would be expected for mosquitoes that have already mated and completed at least one reproductive cycle. Furthermore, when comparing NIRS age predictions with predictions based on the expression of the age-associated gene *SCP1*, we observed a significant positive association for all four models. However, we did observe bias in the age predictions of individual field-collected *Ae. aegypti* for all NIRS models. Thus, while accurate age predictions of individual mosquitoes may not currently be possible using these NIRS models, or for that matter other current age-grading techniques [8], the ability of NIRS to track changes in the population age structure of mosquitoes over time may be a valuable tool in identifying increases in the proportion of older mosquitoes that are more likely to transmit arboviruses.

## Figures and Tables

**Table 1:** Summary data for the generation of the NIRS models. Detailed in this table are the number of mosquitoes utilized for the prediction (Cal.) and cross validation (Test) sets, the spectral range and band regions selected from the model, whether SG1 or multiplicative scatter correction (MSC) was used, the number of factors (Fac), R^2^, slope and 95% confidence interval (CI), intercept (Inter) and 95% CI, standard error of prediction (SEP) and ratio of performance deviation (RPD).

Model Name	Total N	Cal. Set N	Test Set N	Days Tested	Spectral Range	Band Regions	Fac	SG1	MSC	R^2^	Slope (95% CI)	Inter (95% CI)	SEP	RPD
**UGAL lab**	770	609	161	1, 3, 6, 9, 11, 14, 18, 20, 25, 27	500–2400	500-985 1015–2400	11	31	Yes	0.72	0.89 (0.80–0.98)	1.42 (0.02–2.83)	4.56	1.85
**UGAL semi-field**	748	599	149	1, 3, 4, 5, 8, 9, 11, 14, 18	700–2200	700–1850 1910–2200	13	31	Yes	0.66	0.90 (0.80–1.00)	0.98 (0.02–1.94)	2.79	1.70
**Tucson**	540	433	107	1, 3, 4, 5, 6, 7, 8, 9, 12, 13, 14, 16, 18	700–2300	None	14	31	Yes	0.68	0.86 (0.75–0.96)	1.43 (0.02–2.83)	3.15	1.74
**Maricopa**	475	381	94	1, 3, 5, 6, 7, 9, 12, 14, 16, 18	700–2300	None	10	31	Yes	0.79	1.01 (0.90–1.12)	−0.28 (−1.43–0.88)	2.61	2.18

**Table 2:** Linear associations between age predicted by SCP1 analyses and the four NIRS models in 2017 and 2018. Data points with Studentized residuals > |2| were removed before estimating intercepts, slopes and their associated 95% confidence intervals (see Figure 5). N is the number of observations used to fit simple linear regression models. ^1^ For each year, the slopes with different letter had non-overlapping 95% confidence intervals and are considered statistically different (*p* < 0.05).

Year	Model	N	Observations Removed	Intercept (95% CI)	*p*	Slope (95% CI) ^1^	*p*
2017	UGAL lab	127	8	9.60 (7.79–11.40)	<0.0001	0.46 (0.24–0.68) a	<0.0001
	Semi-field	126	8	0.21 (−1.57–1.98)	0.82	0.44 (0.23–0.66) a	<0.0001
	Tucson	128	6	2.32 (0.47–4.17)	0.014	0.27 (0.04–0.50) ab	0.02
	Maricopa	127	7	6.73 (5.63–7.82)	<0.0001	0.08 (−0.05–0.21) b	0.22
2018	UGAL lab	91	5	8.08 (6.55–9.62)	<0.0001	0.48 (0.33–0.63) a	<0.0001
	Semi-field	93	4	1.49 (−0.71–3.70)	0.18	0.48 (0.26–0.70) a	<0.0001
	Tucson	93	4	0.43 (−1.68–2.55)	0.68	0.32 (0.11–0.52) a	0.003
	Maricopa	89	6	7.17 (6.01–8.34)	<0.0001	0.32 (0.21–0.44) a	<0.0001

## Data Availability

Data is contained within the article and supplementary material.

## Supplementary Materials

The following supporting information can be downloaded at: https://www.mdpi.com/article/10.3390/insects13040360/s1, Table S1: Number of *Aedes aegypti* NIRS spectra used to calibrate and test each model at each individual day; Figure S1: Variation in NIRS age predictions in mosquitoes of known age; Data S1: NIRS age predictions, SCP1 age predictions and parity status of individual mosquitoes.

## References

[1] Bowman L.R., Runge-Ranzinger S., McCall P. (2014). Assessing the relationship between vector indices and dengue transmission: A systematic review of the evidence. PLoS Negl. Trop. Dis..

[2] Louis V.R., Phalkey R., Horstick O., Ratanawong P., Wilder-Smith A., Tozan Y., Dambach P. (2014). Modeling tools for dengue risk mapping—A systematic review. Int. J. Health Geogr..

[3] Dye C. (1992). The analysis of parasite transmission by bloodsucking insects. Annu. Rev. Entomol..

[4] Dye C. (1990). Epidemiological significance of vector–parasite interactions. Parasitology.

[5] Dentinova T. (1962). Age-Grouping Methods in Diptera of Medical Importance.

[6] Polovodova V. (1949). The determination of the physiological age of female Anopheles by the number of gonotrophic cycles completed. Medskaya. Parazit..

[7] Hoc T., Charlwood J. (1990). Age determination of *Aedes cantans* using the ovarian oil injection technique. Med. Vet. Entomol..

[8] Hugo L.E., Quick-Miles S., Kay B., Ryan P. (2014). Evaluations of mosquito age grading techniques based on morphological changes. J. Med. Entomol..

[9] Anagonou R., Agossa F., Azondékon R., Agbogan M., Oké-Agbo F., Gnanguenon V., Badirou K., Agbanrin-Youssouf R., Attolou R., Padonou G.G. (2015). Application of Polovodova’s method for the determination of physiological age and relationship between the level of parity and infectivity of Plasmodium falciparum in *Anopheles gambiae s.s*, south-eastern Benin. Parasites Vectors.

[10] Chen C., Mulla M., March R., Chaney J. (1990). Cuticular hydrocarbon patterns in *Culex quinquefasciatus* as influenced by age, sex, and geography. Bull. Soc. Vector Ecol..

[11] Desena M., Clark J., Edman J., Symington S., Scott T., Clark G., Peters T. (1999). Potential for aging female *Aedes aegypti* (Diptera: Culicidae) by gas chromatographic analysis of cuticular hydrocarbons, including a field evaluation. J. Med. Entomol..

[12] Desena M., Edman J., Clark J., Symington S., Scott T. (1999). *Aedes aegypti* (Diptera: Culicidae) age determination by cuticular hydrocarbon analysis of female legs. J. Med. Entomol..

[13] Brei B., Edman J.D., Gerade B., Clark J.M. (2004). Relative abundance of two cuticular hydrocarbons indicates whether a mosquito is old enough to transmit malaria parasites. J. Med. Entomol..

[14] Cook P.E., Hugo L.E., Iturbe-Ormaetxe I., Williams C.R., Chenoweth S.F., Ritchie S.A., Ryan P.A., Kay B.H., Blows M.W., O’Neill S.L. (2006). The use of transcriptional profiles to predict adult mosquito age under field conditions. Proc. Natl. Acad. Sci. USA.

[15] Cook P., Sinkins S. (2010). Transcriptional profiling of *Anopheles gambiae* mosquitoes for adult age estimation. Insect Mol. Biol..

[16] Wang M.-H., Marinotti O., Zhong D., James A.A., Walker E., Guda T., Kweka E.J., Githure J., Yan G. (2013). Gene expression-based biomarkers for *Anopheles gambiae* age grading. PLoS ONE.

[17] Joy T.K., Gutierrez E.H.J., Ernst K., Walker K.R., Carriere Y., Torabi M., Riehle M.A. (2012). Aging field collected *Aedes aegypti* to determine their capacity for dengue transmission in the southwestern United States. PLoS ONE.

[18] Ernst K.C., Walker K.R., Reyes-Castro P., Joy T.K., Castro-Luque A.L., Diaz-Caravantes R.E., Gameros M., Haenchen S., Hayden M.H., Monaghan A. (2017). *Aedes aegypti* (Diptera: Culicidae) longevity and differential emergence of dengue fever in two cities in Sonora, Mexico. J. Med. Entomol..

[19] Weeraratne T.C., Karunaratne S., Reimer L., de Silva W., Wondji C.S. (2021). Use of transcriptional age grading technique to determine the chronological age of Sri Lankan *Aedes aegypti* and *Aedes albopictus* females. Parasites Vectors.

[20] Türker-Kaya S., Huck C.W. (2017). A review of mid-infrared and near-infrared imaging: Principles, concepts and applications in plant tissue analysis. Molecules.

[21] Goh B., Ching K., Soares Magalhães R.J., Ciocchetta S., Edstein M.D., Maciel-de-Freitas R., Sikulu-Lord M.T. (2021). The application of spectroscopy techniques for diagnosis of malaria parasites and arboviruses and surveillance of mosquito vectors: A systematic review and critical appraisal of evidence. PLoS Negl. Trop. Dis..

[22] Mayagaya V.S., Michel K., Benedict M.Q., Killeen G.F., Wirtz R.A., Ferguson H.M., Dowell F.E. (2009). Non-destructive determination of age and species of *Anopheles gambiae sl* using near-infrared spectroscopy. Am. J. Trop. Med. Hyg..

[23] Sikulu M., Killeen G.F., Hugo L.E., Ryan P.A., Dowell K.M., Wirtz R.A., Moore S.J., Dowell F.E. (2010). Near-infrared spectroscopy as a complementary age grading and species identification tool for African malaria vectors. Parasites Vectors.

[24] Sikulu-Lord M.T., Devine G.J., Hugo L.E., Dowell F.E. (2018). First report on the application of near-infrared spectroscopy to predict the age of *Aedes albopictus Skuse*. Sci. Rep..

[25] Sikulu-Lord M.T., Milali M.P., Henry M., Wirtz R.A., Hugo L.E., Dowell F.E., Devine G.J. (2016). Near-infrared spectroscopy, a rapid method for predicting the age of male and female wild-type and Wolbachia infected *Aedes aegypti*. PLoS Negl. Trop. Dis..

[26] Liebman K., Swamidoss I., Vizcaino L., Lenhart A., Dowell F., Wirtz R. (2015). The influence of diet on the use of near-infrared spectroscopy to determine the age of female *Aedes aegypti* mosquitoes. Am. J. Trop. Med. Hyg..

[27] Krajacich B.J., Meyers J.I., Alout H., Dabiré R.K., Dowell F.E., Foy B.D. (2017). Analysis of near infrared spectra for age-grading of wild populations of *Anopheles gambiae*. Parasites Vectors.

[28] Ong O.T., Kho E.A., Esperança P.M., Freebairn C., Dowell F.E., Devine G.J., Churcher T.S. (2020). Ability of near-infrared spectroscopy and chemometrics to predict the age of mosquitoes reared under different conditions. Parasites Vectors.

[29] Williams P., Norris K.H., American Association of Cereal Chemists (2001). Near-Infrared Technology in the Agricultural and Food Industries.

[30] Saeys W., Mouazen A.M., Ramon H. (2005). Potential for onsite and online analysis of pig manure using visible and near infrared reflectance spectroscopy. Biosyst. Eng..

[31] Milali M.P., Kiware S.S., Govella N.J., Okumu F., Bansal N., Bozdag S., Charlwood J.D., Maia M.F., Ogoma S.B., Dowell F.E. (2020). An autoencoder and artificial neural network-based method to estimate parity status of wild mosquitoes from near-infrared spectra. PLoS ONE.

[32] Milali M.P., Sikulu-Lord M.T., Kiware S.S., Dowell F.E., Corliss G.F., Povinelli R.J. (2019). Age grading *An. gambiae* and *An. arabiensis* using near infrared spectra and artificial neural networks. PLoS ONE.

[33] Lambert B., Sikulu-Lord M.T., Mayagaya V.S., Devine G., Dowell F., Churcher T.S. (2018). Monitoring the age of mosquito populations using near-infrared spectroscopy. Sci. Rep..

