# Supplementary material for: Assessing Near-Infrared Spectroscopy (NIRS) for Evaluation of Aedes aegypti Population Age Structure
Source: Insects. 2022 Apr 7;13(4):360. doi: 10.3390/insects13040360 (PMC9029691; doi:10.3390/insects13040360)
Supplement: Supplementary file 1 [file insects-13-00360-s001.zip › Sup. Table 1.pdf]

UGAL lab			UGAL Semi-Field			Tucson			Maricopa		
**Day**	**Calibration**	**Test**	**Day**	**Calibration**	**Test**	**Day**	**Calibration**	**Test**	**Day**	**Calibration**	**Test**
**1**	60	14	**1**	50	12	**1**	34	10	**1**	43	10
**3**	64	17	**3**	42	11	**3**	34	8	**3**	42	11
**6**	45	11	**4**	77	19	**4**	26	7	**5**	49	12
**9**	76	19	**5**	71	17	**5**	43	10	**6**	21	5
**11**	72	18	**8**	84	22	**6**	23	5	**7**	21	5
**14**	48	12	**9**	68	17	**7**	48	12	**9**	42	11
**18**	71	17	**11**	84	20	**8**	24	6	**12**	42	10
**20**	45	12	**14**	78	20	**9**	20	5	**14**	45	11
**25**	75	19	**18**	45	11	**12**	26	7	**16**	28	7
**27**	53	12				**13**	18	5	**18**	47	13
						**14**	43	11			
						**16**	43	11			
						**18**	51	11			

Supplemental Table 1: Number of *Aedes aegypti* NIRS spectra used to calibrate and test each model at each individual day.
