# Supplementary figures and images for: Assessing Near-Infrared Spectroscopy (NIRS) for Evaluation of Aedes aegypti Population Age Structure
Source: Insects. 2022 Apr 7;13(4):360. doi: 10.3390/insects13040360 (PMC9029691; doi:10.3390/insects13040360)

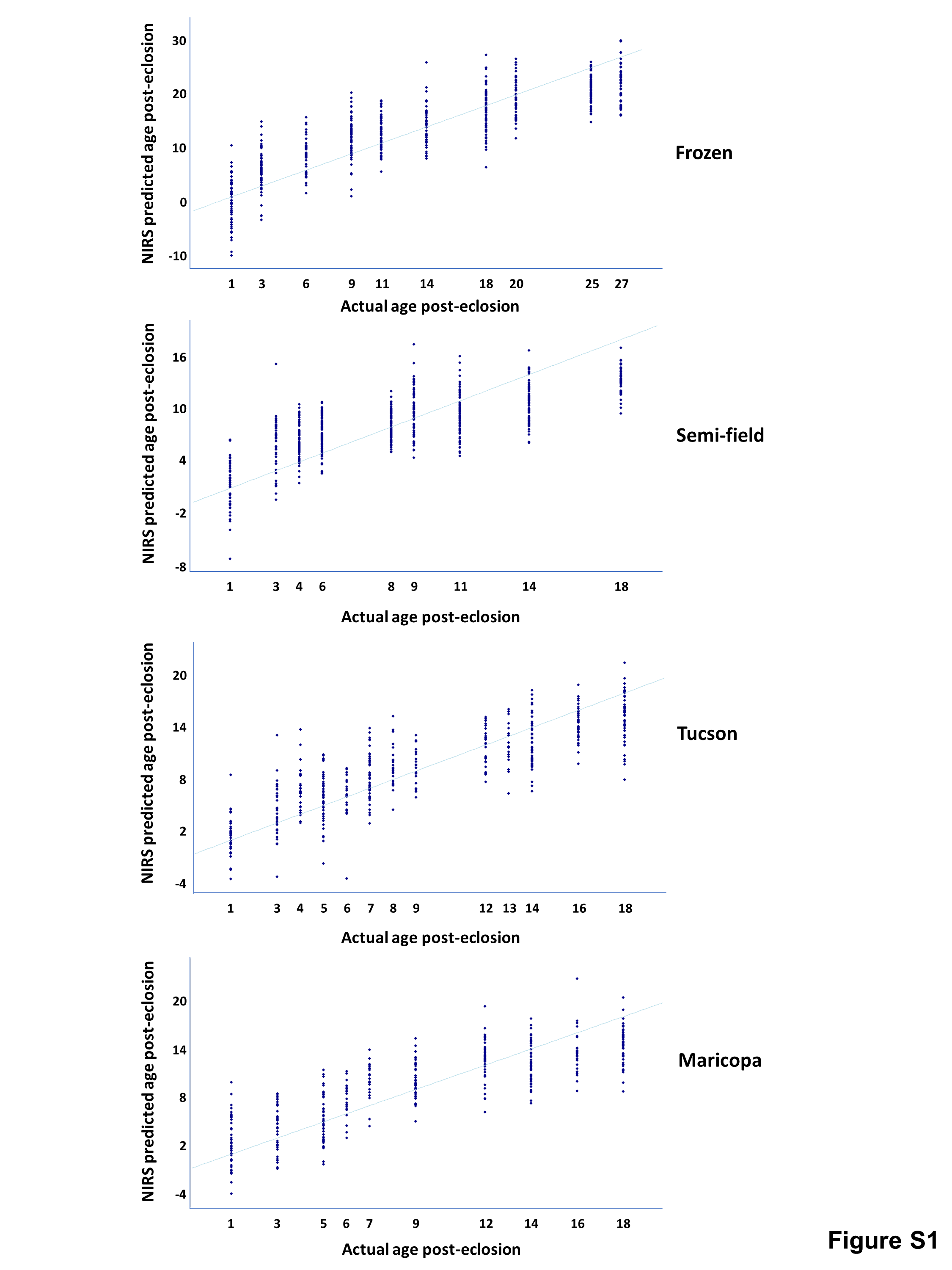

Supplement: Supplementary file 1 [file insects-13-00360-s001.zip › Sup. Figure 1.TIF]
